# Effects of Chaihu-Shugan-San for reflux esophagitis: A protocol for systematic review and meta-analysis

**DOI:** 10.1097/MD.0000000000023458

**Published:** 2020-12-04

**Authors:** Yan Zhou, Zhi Zeng, Xiaoyu Dong, Jianping Fei, Baoliang Li

**Affiliations:** Changzhou Hospital of Traditional Chinese Medicine, Changzhou, China.

**Keywords:** Chaihu-Shugan-San, meta-analysis, protocol, reflux esophagitis, systematic review

## Abstract

**Background:**

Reflux esophagitis (RE) is a common disease which is caused by the reflux of stomach and duodenal contents. As a classic prescription of traditional Chinese medicine, Chaihu-Shugan-San (CSS) has been used in the treatment of RE. However, no critically designed overview to evaluate the systematic review of CSS for RE has been carried out. The purpose of this study is to evaluate the efficacy and safety of CSS in the treatment of RE.

**Methods and analysis:**

We will search the following sources without restrictions for date, language, or publication status: PubMed, Cochrane Central Register of Controlled Trials (CENTRAL) Cochrane Library, EMBASE, MEDLINE, China National Knowledge Infrastructure (CNKI), Wan Fang Database, Chinese Bio-medicine Database, and VIP Chinese Periodical Database. Study selection, data extraction, and assessment of the risk of bias will be performed by 2 reviewers independently. Revman software (v.5.3) will be used to perform the meta-analyses.

**Results:**

This study will provide a comprehensive evaluation of the efficacy and safety of CSS for patients with RE.

**Conclusion:**

The findings will be an available reference to evaluate the efficacy and safety of CSS on RE and provide decision-making reference on which method to choose for clinicians.

**Trial registration number:**

10.17605/OSF.IO/5398R.

## 1 Introduction

Gastroesophageal reflux disease (GERD) is a common disease affecting about 8% to 33% people worldwide.^[1]^ It is characterized by symptoms such as heartburn and acid regurgitation resulting from the reflux of gastric contents back up from the stomach into the esophagus. As a subtype of GERD, reflux esophagitis (RE) is usually caused by the reflux of stomach and duodenal contents, leading to inflammatory lesions in the mucosa of the lower esophagus observed by electronic endoscope.^[2]^ It is a chronic health problem causing troublesome symptoms, affecting quality of life, and bringing a heavy financial burden.^[3,4]^ Thus, effective treatment of RE is of great significance to relieve symptoms and improve the quality of life. At present, proton-pump inhibitors (PPIs) are the standard approach to managing RE patients. However, up to 40% of patients receiving PPI treatment still have reflux symptoms.^[5]^ It is imperative to seek alternative treatments for patients with RE.

Traditional Chinese medicines are widely used in the treatment of digestive diseases since 200 AD in China and has a positive therapeutic effect on GERD.^[6–8]^ As a classic prescription of traditional Chinese medicine recorded in the China Pharmacopoeia (2015 edition), Chaihu-Shugan-San (CSS) has been used in the treatment of functional dyspepsia, chronic gastritis, and RE.^[9–12]^ According to several clinical studies in China, CSS has shown a positive effect on reducing symptoms, improving the quality of life, and reducing the pathological changes of the esophagus on RE.^[12,13]^ However, no critically designed overview to evaluate the systematic review of CSS for RE has been carried out. In this work, we will conduct a systematic review to evaluate the efficacy and safety of CSS in the treatment of RE to provide a reference for clinical application.

## 2 Material and methods

### 2.1 Registration and reporting

The protocol of this systematic review and meta-analysis has been registered in Open Science Framework (OSF, https://osf.io/). The registration DOI of this study is 10.17605/OSF.IO/5398R. The systematic review and meta-analysis (PRISMA) will be referenced throughout the study.^[14]^

### 2.2 Eligibility criteria

#### 2.2.1 Type of studies

Randomized controlled trials (RCTs) which explore the specific efficacy and safety of the CSS in the treatment of RE will be considered for inclusion regardless of publication status and language of publication.

#### 2.2.2 Types of patients

This study will include patients diagnosed with RE by endoscopy and where necessary by pH-impedance monitoring. There will be no limitation about age, sex, region, and other factors. Individuals with other digestive diseases will be excluded.

#### 2.2.3 Type of interventions and comparators

Patients in the treatment group were treated with CSS alone or in combination with conventional pharmacotherapies. The control group included placebo, no treatment, and western medicine recommended by the clinical guidelines.

#### 2.2.4 Types of outcomes

The primary outcomes included the improvement of esophageal histopathology, overall efficiency, reflux disease diagnostic questionnaire (RDQ) score, and symptom total score. And the secondary outcomes included relapse rate and adverse reactions.

### 2.3 Information source and search strategy

To identify all related studies, we will search the following sources without restrictions for date, language, or publication status: PubMed, Cochrane Central Register of Controlled Trials (CENTRAL) Cochrane Library, EMBASE, MEDLINE, China National Knowledge Infrastructure (CNKI), Wan Fang Database, Chinese Bio-medicine Database, and VIP Chinese Periodical Database. A combination of Medical Subject Heading (MeSH) and free-text terms will be applied to implement search strategies. The search strategy in PubMed was as follows:

1#: Search: ((chaihu shugan san[MeSH Terms]) OR (chaihu shugan power[Title/Abstract])) OR (chaihu shugan[Title/Abstract]).

2#: Search: ((((((esophagitis[MeSH Terms]) OR (esophagitis, peptic[MeSH Terms])) OR (gastroesophageal reflux[MeSH Terms])) OR (reflux esophagitis[Title/Abstract])) OR (gastric acid reflux[Title/Abstract])) OR (acid reflux, gastric[Title/Abstract])) OR (gastro esophageal reflux[Title/Abstract]).

3#:((((((((clinical trials, randomized[MeSH Terms]) OR (randomized controlled trial[MeSH Terms])) OR (controlled clinical trials, randomized[MeSH Terms])) OR (RCT[Title/Abstract])) OR (controlled clinical trial[Title/Abstract])) OR (randomized[Title/Abstract])) OR (trial[Title/Abstract]).

#1 and #2 and #3

### 2.4 Data collection and analysis

#### 2.4.1 Study selection

**Figure 1:**
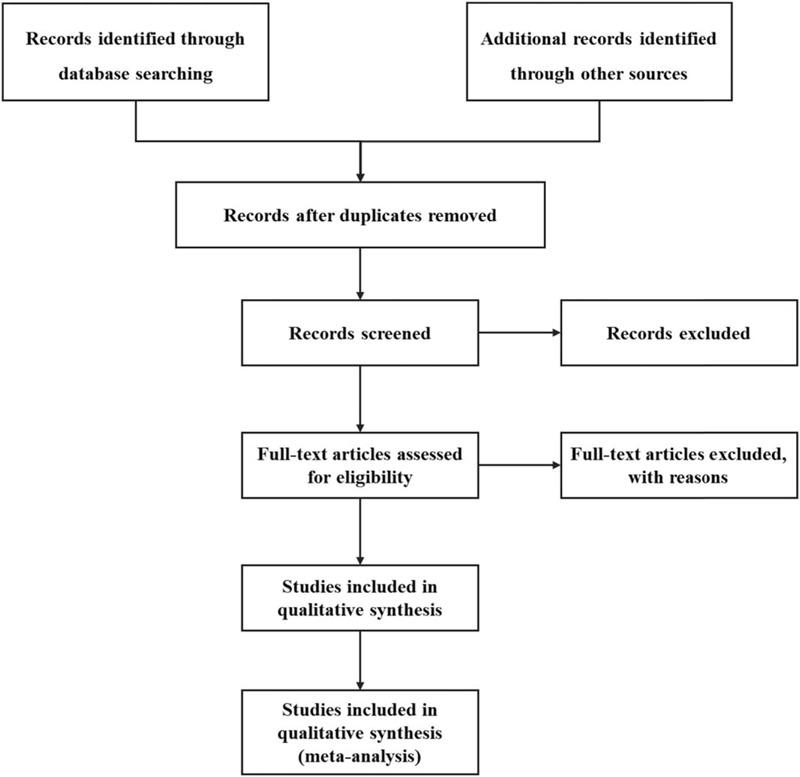
Flow chart of study selection.

Two reviewers will independently perform literature screening, study selection, and data extraction based on the research criteria and search strategies introduced above. The articles will be imported into EndnoteX9 (Stanford, Connecticut, https://endnote.com) to screen the title and abstract, the duplications. The eligible articles will be further determined for inclusion by reading the full text. Any disagreements generated between the 2 reviewers will be resolved through discussion with other reviewers. The details of the selection process are shown in Fig. 1.

#### 2.4.2 Data extraction and management

Two authors will independently extract data from included studies. Any disagreement will be resolved by discussion or by consultation with a third author. The characteristic information including the first author's name, year of publication, study methodology, severity, intervention and control, sample size, duration of intervention, and outcomes will be export to a predesigned data extraction form. A third reviewer will validate data. The corresponding author of original RCT will be contacted if data are missing or unclear.

#### 2.4.3 Assessment of risk of bias

The methodological quality of eligible studies will be assessed according to the Cochrane Handbook for Systematic Reviews of Interventions. The risk of bias of a trial will be evaluated through 7 items, including random sequence generation (selection bias), allocation concealment (selection bias), blinding of participants and personnel (performance bias), blinding of outcome assessment (detection bias), incomplete outcome data (attrition bias), selective reporting (reporting bias), other bias. The studies will be evaluated as “Low risk,” “High risk,” or “Unclear risk.”

#### 2.4.4 Data synthesis

The RevMan 5.3 (Cochrane, London, UK) software will be used for data analysis. For dichotomous outcomes, we will conduct a random effects meta-analysis with risk ratios and report 95% confidence intervals. For continuous outcomes, the mean difference or standard MD with 95% CIs will be utilized for evaluating the treatment effect.

#### 2.4.5 Heterogeneity investigation

We will assess statistical heterogeneity using Cochrane *X*^*2*^ and *I*^*2*^ tests.^[15]^ A fixed effect model will be used if there is no obvious heterogeneity (*P* ≥ .1 and *I*^2^ ≤ 50%). A random effects model being used if significant heterogeneity is found to exist (*P* < .1 and *I*^2^ > 50%).

#### 2.4.6 Subgroup analysis and sensitivity analysis

If the necessary data are available, subgroup analyses will be conducted for the different types of control group, duration, and severity of disease at baseline. Sensitivity analysis will be applied to determine the robustness of the results by ruling out studies of low quality and small sample size.

#### 2.4.7 Assessment of publication bias

If there are >10 trials included in the study, publication bias will be analyzed by visual inspection of funnel plots.

#### 2.4.8 Grading the quality of evidence

The quality of evidence will be evaluated using the Grading of Recommendations Assessment, Development, and Evaluation (GRADE).^[16]^ The quality of evidence will be categorized into 4 levels: high, moderate, low, and very low quality.

#### 2.4.9 Ethics and dissemination:

This is a meta-analysis study based on previously published data, so patient and public involvement will not be included in this study. This systematic review will not require ethical approval because there are no data used in our study that are linked to individual patient data.

## 3 Discussion

As one of the most common gastrointestinal diseases, RE has attracted increased attention owing to its growing burden and negative impact on quality of life. Thus, effective intervention should be conducted in the treatment of RE. Over the past few decades, a number of RCTs have been conducted to evaluate the effectiveness and safety of CSS on the treatment of RE, it is necessary to summarize and evaluate these studies. This meta-analysis will provide a relatively convincing conclusion of whether CSS is effective for patients with RE. The results of our work will provide helpful information for clinicians and patients to treat RE.

## Author contributions

**Conceptualization:** Yan Zhou.

**Data curation:** Zhi Zeng, Xiaoyu Dong, Jianping Fei.

**Formal analysis:** Zhi Zeng, Xiaoyu Dong.

**Investigation:** Jianping Fei.

**Methodology:** Jianping Fei.

**Software:** Yan Zhou, Zhi Zeng.

**Supervision:** Baoliang Li.

**Validation:** Baoliang Li.

**Writing – original draft:** Yan Zhou.

**Writing – review & editing:** Zhi Zeng, Xiaoyu Dong.
